# CEMIP (KIAA1199) induces a fibrosis-like process in osteoarthritic chondrocytes

**DOI:** 10.1038/s41419-019-1377-8

**Published:** 2019-02-04

**Authors:** Céline Deroyer, Edith Charlier, Sophie Neuville, Olivier Malaise, Philippe Gillet, William Kurth, Alain Chariot, Michel Malaise, Dominique de Seny

**Affiliations:** 1 Laboratory of Rheumatology, GIGA I3, CHU de Liege, University of Liege, Liege, Belgium; 2 Orthopedic Surgery Unit, CHU of Liege, Belgium; 3 Laboratory of Medical Chemistry, GIGA Molecular Biology of Diseases, University of Liege, Liege, Belgium; 4 Walloon Excellence in Life Sciences and Biotechnology (WELBIO), Liege, Belgium

## Abstract

CEMIP (for “Cell migration-inducing protein” also called KIAA1199 and Hybid for “Hyaluronan-binding protein”) expression is increased in cancers and described as a regulator of cell survival, growth and invasion. In rheumatoid arthritis, CEMIP is referred to as an angiogenic marker and participates in hyaluronic acid degradation. In this study, CEMIP expression is investigated in healthy and osteoarthritis (OA) cartilage from human and mouse. Its role in OA physiopathology is deciphered, specifically in chondrocytes proliferation and dedifferentiation and in the extracellular matrix remodeling. To this end, CEMIP, αSMA and types I and III collagen expressions were assessed in human OA and non-OA cartilage. CEMIP expression was also investigated in a mouse OA model. CEMIP expression was studied in vitro using a chondrocyte dedifferentiation model. High-throughput RNA sequencing was performed on chondrocytes after CEMIP silencing. Results showed that CEMIP was overexpressed in human and murine OA cartilage and along chondrocytes dedifferentiation. Most of genes deregulated in CEMIP-depleted cells were involved in cartilage turnover (e.g., collagens), mesenchymal transition and fibrosis. CEMIP regulated β-catenin protein level. Moreover, CEMIP was essential for chondrocytes proliferation and promoted αSMA expression, a fibrosis marker, and TGFβ signaling towards the p-Smad2/3 (Alk5/PAI-1) pathway. Interestingly, CEMIP was induced by the pSmad1/5 (Alk1) pathway. αSMA and type III collagen expressions were overexpressed in human OA cartilage and along chondrocytes dedifferentiation. Finally, CEMIP was co-expressed in situ with αSMA in all OA cartilage layers. In conclusion, CEMIP was sharply overexpressed in human and mouse OA cartilage and along chondrocytes dedifferentiation. CEMIP-regulated transdifferentiation of chondrocytes into “chondro-myo-fibroblasts” expressing α-SMA and type III collagen, two fibrosis markers. Moreover, these “chondro-myo-fibroblasts” were found in OA cartilage but not in healthy cartilage.

## Introduction

CEMIP for “Cell migration-inducing protein” (also called KIAA1199 and Hybid), was originally detected in the inner ear and reported as the cause of nonsyndromic hearing loss^1,2^. The increase of CEMIP expression was also observed in various cancers^3,4^, and described as a key regulator of cell survival, growth and invasion^5,6^. Moreover, CEMIP expression was also enhanced in human papillomavirus (HPV) infection and characterized as an EGFR-binding protein that promotes EGF-mediated epithelial–mesenchymal transition (EMT)^6^. CEMIP is involved in the Wnt/β-catenin signaling pathway^3,7^ as well as in the enhanced degradation of hyaluronic acid (HA) in dermal fibroblast^8^. Furthermore, CEMIP is increased in synovial fibroblasts from patients with osteoarthritis (OA) and rheumatoid arthritis (RA) and is detected in the synovium of RA patients and referred to as an angiogenic marker^8,9^. Recently, a role of CEMIP in endochondral ossification has been highlighted^10^. Up to now, the role of CEMIP in OA chondrocytes remains unknown.

OA is a degenerative disease affecting the entire joint. It is mainly characterized by cartilage degradation, synovial inflammation, subchondral bone erosion, and osteophyte formation. In OA, anabolic capacity of chondrocytes is largely decreased, thus impairing cartilage repair. In an advanced stage, chondrocytes dedifferentiate into fibrochondrocytes producing abnormal components such as fibronectin fragments^11^. Ultimately, there is a reset of the cell cycle leading to chondrocyte proliferation, hypertrophy and finally cell death by apoptosis^12^. The newly acquired proliferative activity of chondrocytes is often observed by clustering features in OA cartilage^12^. In sum, a catabolic hyperactivity followed by a default of anabolic response and chondrocyte dedifferentiation/proliferation/apoptosis contributes to the degradation of the extracellular cartilage matrix in OA cartilage.

Several chondrocyte phenotypes can be depicted in cartilage according to their collagen expression profile and their localization inside cartilage^12^. Activated chondrocytes synthesize collagen type II, IX, and XI, and are present in the middle zone of cartilage^13,14^. Hypertrophic chondrocytes rather express collagen type X and are found in the deepest zones of cartilage^15^. Chondrocytes expressing collagen type I and III are located in the upper middle zone of OA cartilage^12,16^, and could be related to the so-called dedifferentiation process resulting from a modulation of the chondrocyte phenotype to a fibroblast-like phenotype. Recently, we highlighted that in vitro spontaneous dedifferentiated chondrocytes are able to express OA-related protein such as collagen type I, β-catenin, and leptin, in contrast to freshly isolated chondrocytes. Inversely, collagen type II and X and Sox-9 are expressed in freshly isolated chondrocytes but nearly not in dedifferentiated chondrocytes^17^. In human normal cartilage, type II collagen is the main collagen type present while type X collagen is expressed by hypertrophic chondrocytes in OA cartilage^18^.

In the present study, the expression of CEMIP is for the first time investigated in the cartilage of humans and mice. The role of CEMIP in cellular mechanisms leading to OA is also unraveled specifically in cell proliferation, in chondrocyte dedifferentiation and ECM remodeling but also in newly acquired functions as pro-fibrotic mediator.

## Material and methods

### Subject recruitment

Tissues collection was made in collaboration with the Musculoskeletal Surgery department (CHU Sart-Tilman, ULiege). OA cartilage was obtained from 64 patients (41 females and 23 males) undergoing knee (*n* = 49) or hip (*n* = 15) replacement surgery, mean age was 67 (range 22–91) years and mean BMI was 28.35 (range 11.97–41.4) kg/m^2^. Healthy cartilage was obtained from 9 patients suffering from subcapital femoral neck fracture (5 females and 4 males). Mean age was 79 (range 65–90) years and mean BMI was 22.83 (range 17.99–25.71) kg/m^2^. The research ethics committee of CHU de Liege, Belgium, approved the study and verbal informed consent was obtained to allow research procedures on the tissues collected, as explained in the institutional information booklet written by the hospital and provided to each patient.

### Animal experimentation procedures

All experiments were approved by the local ethical committee (#14–1721 University of Liege, Liege, Belgium). 10-weeks-old C57BL6 male mice were used for the study. The DMM and sham surgeries were performed on knees following the Glasson et al.^19^ protocol. Briefly, mice were anesthetized using isoflurane and knees were sanitized prior surgery. Surgery was performed using a surgical microscope. A longitudinal incision from the distal patella to the proximal tibial plateau was made. The joint capsule was incised from the distal patella to the proximal tibial plateau to expose the meniscotibial ligament of the medial meniscus. Sham surgery was stop at this point. For destabilization of the medial meniscus (DMM surgery) the medial meniscotibial ligament was sectioned using a fine steel surgical blades (SM65, Swann-Morton, Sheffield, United Kingdom). The joint capsule was closed with resorbable 10–0 VicrylTM (V971G, Ethicon, Somerville, New Jersey, USA) and the skin was closed with resorbable 8–0 VicrylTM (V400G, Ethicon). All mice received a cocktail of Enrofloxacin (10 mg/kg) and Buprenophrin (0.09 mg/kg), preoperatively two times a day, 1 day before and 3 days post-surgery. Mice were sacrificed 4, 8, 12, 20, or 24 weeks after surgery for time-course analysis. Quantitative analyzes were performed on mice killed 4 and 8 weeks after surgery. OA score was quantified using the modified Pritzker OARSI score^20^.

### Immunohistochemistry analysis

IHC analyzes were performed on cartilage tissues obtained from human hip fractures, OA hips and OA knees and from sham and OA mice. Tissues were fixed in 4% paraformaldehyde for 24 h, decalcified in DC2 (Labonord, Templemars, France) for 24 h for human sample and in EDTA for 15 days for mouse samples, dipped in 70% (v/v) ethanol and embedded in paraffin. Immunohistochemistry was performed on cartilage tissue sections (5μm) after dewaxing and chondroitinase unmasking using a primary monoclonal antibody against CEMIP (Santa Cruz, Dallas, Texas, USA for human cartilage and from Proteintech, Rosemont, Illinois, USA for mice sections), against αSMA (Agilent, Santa Clara, California, USA), against type I collagen (Abcam, Cambridge, Massachusetts, USA), against type III collagen (Abcam) or against type X collagen (Invitrogen, Carlsbad, Californie, USA) were used. Sections were then incubated with Envision + System-HRP (Agilent, Santa Clara, California, USA). Peroxidase was detected with Liquid DAB + Substrate Chromogen System (Agilent). Sections were finally counterstained with Carazzy’s Hematoxylin (EMD Millipore, Billerica, Massachusetts, USA). Sections incubated without primary antibody served as controls. Staining was revealed with Nanozoomer Digital Pathology 2.0 HT scanner (Hamamatsu photonics, Hamamatsu, Japan). The number of CEMIP, αSMA, and type I and III collagen-positive cells was counted blindly by two different human operators with the cytomine software (http://www.cytomine.be/)^21^.

### Cell culture and reagents

Human chondrocytes and synoviocytes were isolated from knee joint as previously described^22^. Cells were cultured in DMEM medium supplemented with 10% fetal bovine serum, 1% l-glutamine (200 mM), 100 units/ml penicillin and 100 μg/ml streptomycin (BioWhittaker, Walkersville, Maryland, USA). Cells were maintained at 37 °C in a 5% CO_2_ atmosphere. For in vitro dedifferentiated model, freshly isolated chondrocytes were cultivated in monolayer during 1, 4, or 14 days. TGFβ (Sigma-Aldrich, Saint Louis, Missouri, USA) 10 ng/ml and SB431542 (Sigma-Aldrich) 1.5 μM were employed on dedifferentiated chondrocytes.

### Lentiviral cell infection

The lentiviral vectors (rLV) were generated with the help of the GIGA-Viral vector Plateform. Briefly, Lenti-X 293T cells (Clontech, Mountain view, Clifornie, USA, 632180) were co-transfected with a pSPAX2 (Addgene, Cambridge, Massachusetts, USA, Plasmid #12260) and a VSV-G encoding vector. Pseudotype formation of murine leukemia virus with the G protein of vesicular stomatitis virus along with CEMIP shRNAs plasmids (Sigma-Aldrich, #1 TRCN0000118791, #2 TRCN0000118787), SMAD1 shRNA plasmid (Sigma-Aldrich, TRCN0000423087) or with a control sequence directed against eGFP (Sigma, Belgium, SHC005) encoding plasmid^23^. 48 and 72 h post transfection, viral supernatants were collected, filtrated and concentrated 100× by ultracentrifugation. The lentiviral vectors were then titrated with qPCR Lentivirus Titration (Titer) Kit (ABM, USA, LV900). After chondrocytes transduction with lentiviral vectors, supernatants were harvested and whole-cell lysates or RNA extractions were performed.

Total RNAs from articular chondrocytes were extracted using Nucleospin RNA kit (Macherey-Nagel, Düren, Germany). Reverse transcription was performed with RevertAid H Minus First Strand cDNA Synthesis Kit (Thermo Scientific, Pittsburgh, Pennsylvania, USA) according to the manufacturer’s instructions. cDNA products were then amplified using Real-time reverse transcription quantitative PCR (RT-qPCR) with the KAPA SYBR FAST detection system (Sopachem, Eke, Belgium) and experiments were run on a LightCycler 480 instrument (Roche Diagnostics, Mannheim, Germany). Data were analyzed using LC480 software release 1.5.0 SP4. For each gene, cDNA dilution curves were generated and used to calculate the individual real-time PCR efficiencies (*E* = 10(−1/slope)). The 2^−^^ΔΔCT^ method was used to calculate the relative gene expression between freshly isolated (calibrator sample) and dedifferentiated chondrocytes or between shEGFP transduced chondrocytes (calibrator sample) and shCEMIP transduced chondrocytes. Input amounts were normalized with the GAPDH endogenous control gene. Primers were purchased from Eurogentec (Seraing, Belgium) or Integrated DNA Technologies (Coralville, Iowa, USA).

### RNA-sequencing analysis

The Illumina Truseq stranded mRNA Sample Preparation kit was used to prepare libraries from 500 nanograms of total RNAs according to manufacturer protocol. RNA integrity was verified on the Bioanalyser 2100 with RNA 6000 Nano chips and RIN scores were >9 for all samples. Libraries were validated on the Bioanalyser DNA 1000 chip and quantified by qPCR using the KAPA library quantification kit. All libraries were sequenced on an Illumina NextSeq500 sequencer in a single run generating minimum 26 millions single-end 76 base reads per library. Library quality was successfully confirmed by FastQC and Picard analyses. Reads were aligned to UCSC hg19 human reference genome with Star aligner. Differential expression analysis was performed at the gene level with DESeq2 comparing samples corresponding to the same patient (paired analysis) between shEGFP and either shCEMIP#1 or #2^24^. Genes significantly up or down-regulated (False discovery rate ≤ 10%) for both comparisons were selected for Gene set enrichment analysis (GSEA, http://www.broadinstitute.org/gsea).

### Western blot analysis

Protein extracts were prepared by lysing the chondrocytes in Rippa buffer. Proteins were separated by SDS-PAGE and transferred to polyvinylidene difluoride membranes (EMD Millipore). After blocking, membranes were incubated with anti-CEMIP (Phoenix Pharmaceutical, Burlingame, CA, USA), anti-β-catenin and HSP90 (Santa Cruz Technologies) and anti-β-TRCP, αSMA, SMAD1, p-Smad2, and pSmad1/5 (Cell Signaling, Beverly, Massachusetts, USA). Membranes were then incubated with 1:1000 diluted peroxidase-conjugated anti-mouse or anti-rabbit secondary antibodies (Cell Signaling). Reactions were revealed with the enhanced chemiluminescence detection reagent (ECL kit, Thermo Fisher Scientific, Waltham, Massachusetts USA). Detected signals were analyzed by densitometry. The intensity of each band was measured with Image Studio Lite Software (Li-Cor Biosciences, Linkolin, Nebraska, NE). To normalize protein levels, the value of the band corresponding to each protein level was normalized with the intensity of the corresponding anti-HSP90 signal used as an internal standard.

### Statistical analysis

For two groups comparisons, non-parametric Mann–Whitney test or non-parametric Wilcoxon-paired test were used. For multiple comparisons, a log-transform was applied to all variables to normalize their distribution. Data were analyzed by paired or unpaired ANOVA test, followed by Tukey post hoc test. Calculations and graphs (Mean with SEM) were done with GraphPad prism software (version 5.0, La Jolla, California, USA). Results were considered significant at the 5% critical level (*p < 0.05, **p < 0.01, ***p < 0.001).

## Results

### CEMIP expression in mouse and human OA cartilage and in human chondrocytes

**Fig. 1:**
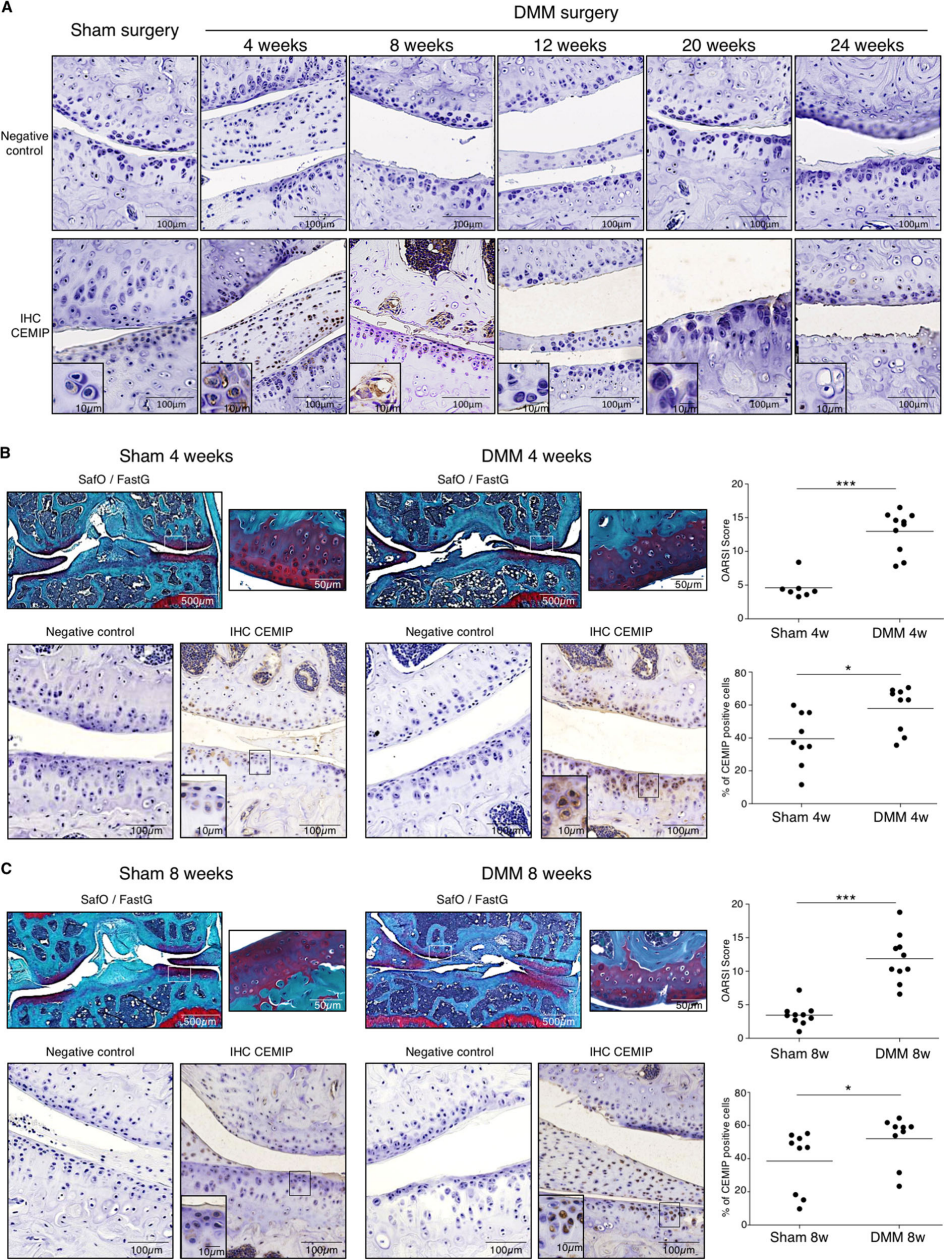
CEMIP expression is increased in mouse OA cartilage. **a** Representative IHC picture of cartilage section from sham and DMM mice at 4, 8, 12, 20, and 24 weeks stained without (negative control) and with anti-CEMIP antibody. **b** Representative picture of cartilage section from sham and DMM mice at 4 weeks stained with SafraninO-FastGreen. OA score was significantly increased in DMM mice (*n* = 10) compared to Sham mice (*n* = 7) (up). Representative IHC picture of cartilage section stained without (negative control) and with an anti-CEMIP antibody. Analysis was done on several mice (*n* = 9 for each group): the percentage of CEMIP positive chondrocytes was significantly increased in DMM mice at 4 weeks compared to sham mice (bottom). (Mann–Whitney test: *p < 0.05 and **p < 0.01). **c** Representative picture of cartilage section from sham and DMM mice at 8 weeks stained with SafraninO-FastGreen. OA score was significantly increased in DMM mice compared to Sham mice (*n* = 10 for each group) (up). Representative IHC picture of cartilage section stained without (negative control) and with an anti-CEMIP antibody. Analysis was done on several mice (*n* = 9 for each group): the percentage of CEMIP positive chondrocytes was significantly increased in DMM mice at 8 weeks compared to sham mice (bottom). (Mann–Whitney test: *p < 0.05 and **p < 0.01)

CEMIP immunohistochemistry (IHC) analysis was performed on the cartilage of sham and OA mice at different stages of the disease. After DMM surgery, mice were first killed at various intervals of time during 24 weeks and CEMIP expression was analyzed every 4 weeks. The expression of CEMIP was increased at 4 and 8 weeks post-surgery compared to sham mice but not in the late stages of the disease (at 12, 20, and 24 weeks post-surgery) (Fig. 1a). IHC quantification was then performed on tibia and femur of DMM mice after 4 and 8 weeks of surgery and CEMIP expression was significantly increased at both time in the cartilage of DMM mice compared to healthy cartilage of sham mice (Fig. 1b, c). SafraninO-FastGreen coloration, OA quantification (OARSI score), and representative images of IHC negative controls are presented in the figure.

**Fig. 2:**
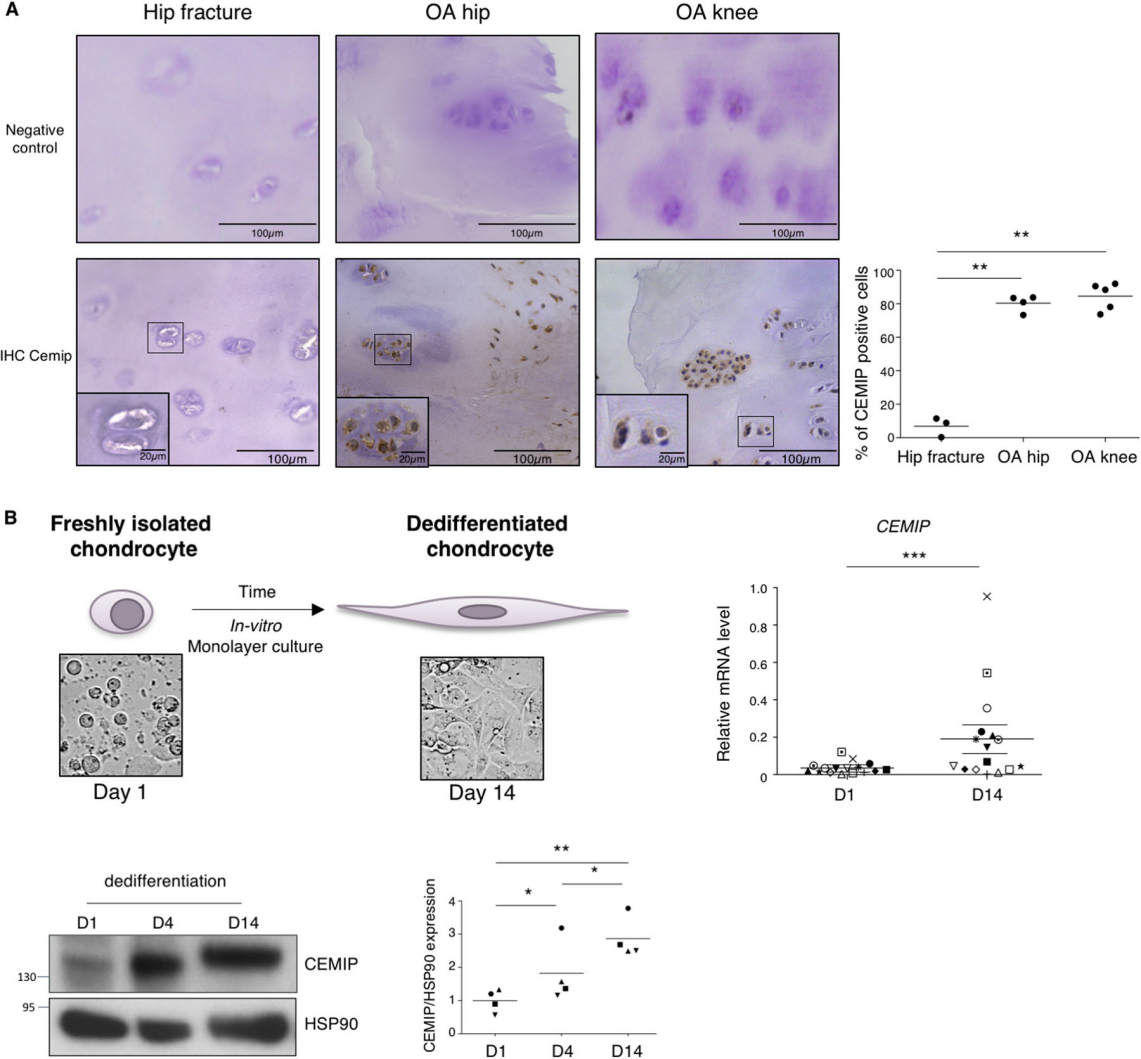
CEMIP expression is increased in human OA cartilage and in human dedifferentiated chondrocytes. **a** Representative IHC picture of cartilage section from hip fracture, OA hip and OA knee stained without (negative control) and with an anti-CEMIP antibody (Left). Analysis was done on several patients: the percentage of CEMIP positive cells was significantly increased in OA hip (*n* = 4) and OA knee (*n* = 5) compared to hip fracture (*n* = 3) (right). (ANOVA tests: **p < 0.01). **b** Schematic representation of in vitro chondrocyte dedifferentiation (top left). *CEMIP* mRNA levels were significantly increased at D14 compared to D1 (*n* = 16) (error bar: SED, Wilcoxon-paired test: *p* < 0.001) (top right). Representative picture of western blot using CEMIP and HSP90-specific antibodies at D1, D4, and D14 is presented (bottom left). Analysis was done on several patients (*n* = 4): western blot quantification illustrating the significant increase of CEMIP/HSP90 expression at D14 compared to D1 and D4 and at D4 compared to D1 (bottom right). (ANOVA paired tests: *p < 0.05, **p < 0.01)

CEMIP expression was also investigated in human cartilage. After quantification, CEMIP expression was significantly increased in OA human hip and knee compared to non-OA human hip cartilage (Fig. 2a). Representative images of IHC negative controls are shown in the figure.

Finally, CEMIP expression was studied, in vitro, in the dedifferentiation model of chondrocytes isolated from OA human knee cartilage. The dedifferentiation process was validated by assessing the increased expression of *COL1A1* mRNA and β-catenin protein level as previously described^17^ (supplementary data 1). *CEMIP* mRNA and protein levels were detected in freshly isolated chondrocytes and were significantly increased at day 14 (D14) compared to day 1 (D1) (Fig. 2b).

### Identification of CEMIP target genes in human dedifferentiated chondrocytes

**Fig. 3:**
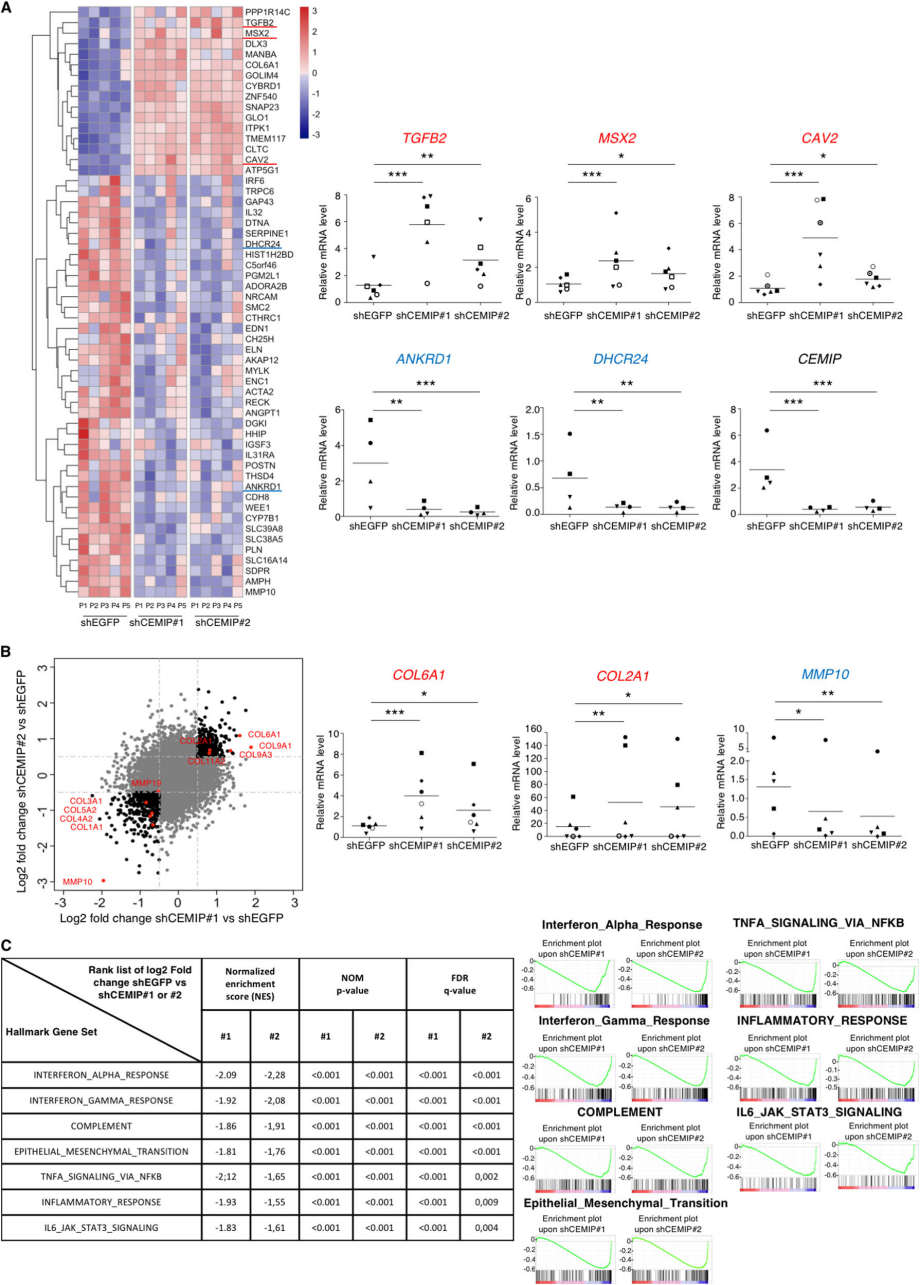
CEMIP modulates ECM components in human dedifferentiated chondrocytes. **a** Heatmap of differential gene expression values upon CEMIP depletion in chondrocytes from 5 patients for genes with a shrunk Log2 fold change better than ±1 (left). RT-qPCR analysis of *TGFB2*, *MSX2*, *CAV2*, *ANKRD1*, *DHCR24*, and *CEMIP* genes in CEMIP-depleted chondrocytes compared to non-depleted chondrocytes. *TGFB2* (*n* = 6), *MSX2* (*n* = 6), and *CAV2* (*n* = 6) relative gene expressions were increased in CEMIP-depleted cells compared to non-depleted cells while *ANKRD1* (*n* = 4), *DHCR24* (*n* = 4), and *CEMIP* (*n* = 6) were decreased (right). (ANOVA paired tests: *p < 0.05, **p < 0.01, ***p < 0.001). **b** Dot plot representing the comparison of Shrunk Log2 fold change for shCEMIP#1 compared to shEGFP and shCEMIP#2 compared to shEGFP. Genes with an adjusted *p*-value < 0.01 and a Shrunk Log2 fold change < −0.5 or >0.5 for both comparisons are represented in black. Genes of ECM components are highlighted in red (left). RT-qPCR analysis of different ECM component genes. *COL6A1* (*n* = 6), *COL2A1* (*n* = 6) relative genes expressions were increased in CEMIP-depleted cells compared to non-depleted cells while *MMP10* (*n* = 5) relative gene expression was decreased (right). (ANOVA paired tests: *p < 0.05, **p < 0.01, ***p < 0.001). **c**. GSEA analysis using Hallmark pathway database. NES and adjusted *p*-values are added for each enrichment dataset and for both comparisons (shCEMIP#1 vs shEGFP and shCEMIP#2 vs shEGFP)

To extensively identify all signaling pathways that rely on CEMIP expression, the transcriptome of human OA knee dedifferentiated chondrocytes was profiled in the presence or absence of CEMIP expression (using 2 specific shRNAs). 2675 genes were significantly down or up-regulated in CEMIP-depleted cells (with both shRNAs) compared to control cells (*p* < 0.001). Among them, 56 had a shrunk Log2 fold change higher than ±1 (Fig. 3a, left). The increased expression of three genes (*TGFB2*, *MSX2*, and *CAV2*) and the decrease expression of two genes (*ANKRD1* and *DHCR24*) described as being related to OA pathology^25–28^ were confirmed with another set of patients by RT-qPCR (Fig. 3a, right). Among the 56 genes, several were involved in OA cartilage metabolism (Fig. 3b, left). Indeed, there was a decrease of *MMP10*, *COL1A1*, *COL3A1*, *COL4A2, COL5A2* and MMP19 and an increase of *COL2A1, COL6A1*, *COL9A1, COL9A3*, and *COL11A2* mRNA levels in CEMIP-depleted cells, compared to shEGFP control cells (supplementary data 2). On another set of patients, the increase of *COL2A1* and *COL6A1* and the decrease of *MMP10* mRNA level upon CEMIP deficiency were confirmed by RT-qPCR (Fig. 3b, right). The decreased expression of *COL1A1* and *COL3A1* is further described below and illustrated in Fig. 5a.

These data suggest that CEMIP is involved in the cartilage turnover process.

### CEMIP regulated a mesenchymal transition process

**Fig. 4:**
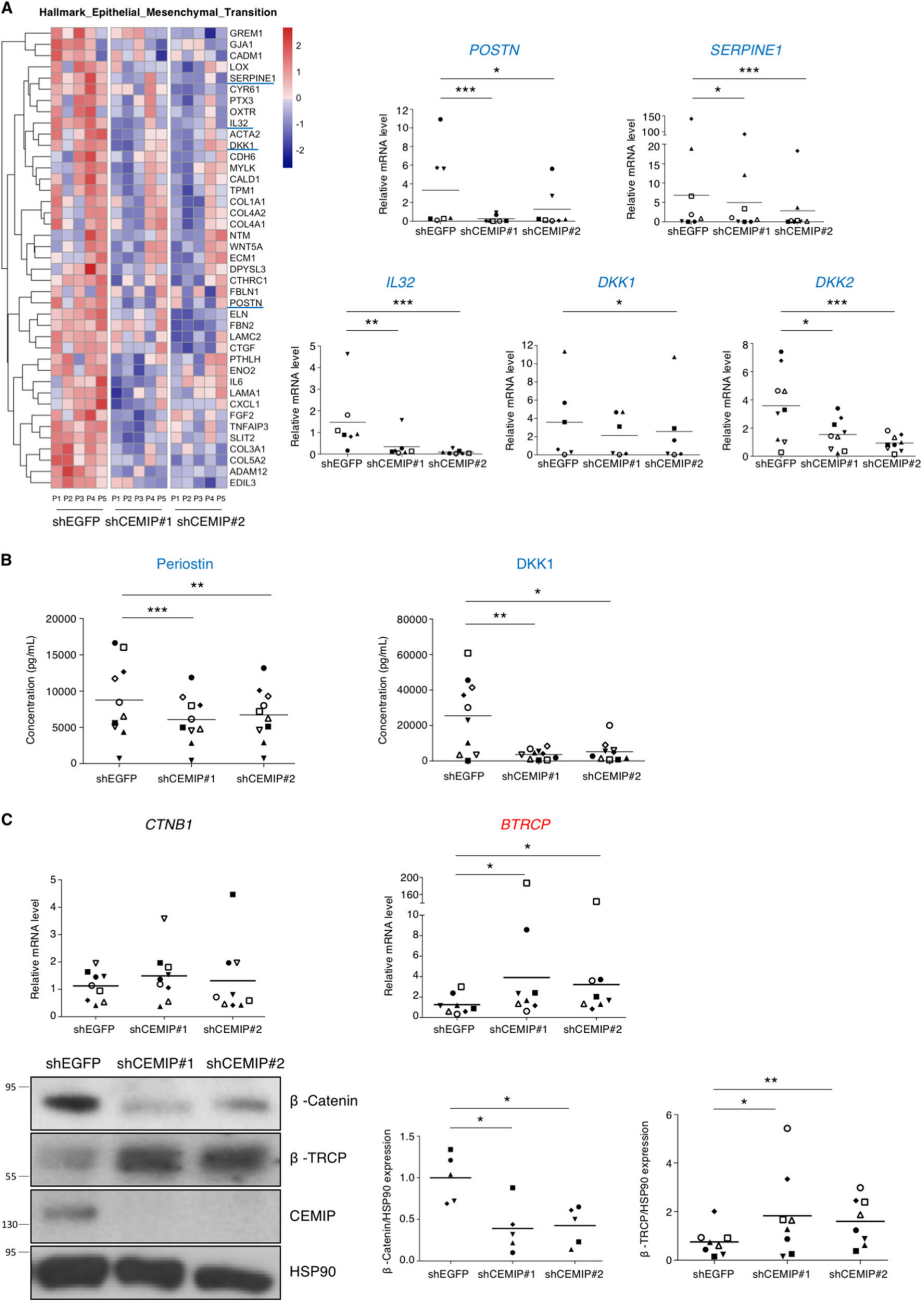
CEMIP modulated a mesenchymal transition-like process in human dedifferentiated chondrocytes. **a** The EMT pathway highlighted by GSEA analysis and the corresponding heatmap after CEMIP depletion in chondrocytes from five patients (left). RT-qPCR analysis of *POSTN*, *SERPINE1*, *IL32*, *DKK1*, and *DKK2*, genes in CEMIP-depleted chondrocytes compared to non-depleted chondrocytes. *POSTN* (*n* = 7), *SERPINE1* (*n* = 8), *IL32* (*n* = 7), *DKK1* (*n* = 6), and *DKK2* (*n* = 9) relative gene expressions were decreased in CEMIP-depleted cells (right). (ANOVA paired tests: *p < 0.05, **p < 0.01, ***p < 0.001). **b** ELISA analysis of Periostin (left) and DKK1 (right) secretion in CEMIP-depleted cells and control cells. Secretions of Periostin (*n* = 10) and DKK1 (*n* = 10) were decreased in CEMIP-depleted chondrocytes compared to control chondrocytes. (ANOVA paired tests: *p < 0.05, **p < 0.01, ***p < 0.001). **c** RT-qPCR analysis of CTNB1 and BTRCP genes in CEMIP-depleted chondrocytes compared to non-depleted chondrocytes (up). BTRCP (*n* = 8) was increased in CEMIP-depleted cells compared to control cells while CTNB1 (*n* = 9) expression was not modified (ANOVA paired tests: *p < 0.05). Representative picture of western blot analysis of β-catenin, β-TRCP, CEMIP, and HSP90 expression in cells treated with shEGFP, shCEMIP#1, and shCEMIP#2 (left). Analysis was done on several patients: western blot quantification illustrating the decrease of β-catenin/HSP90 expression (*n* = 5) and the increase of β-TRCP/HSP90 (*n* = 8) in cells treated with shCEMIP#1 and #2 compared to shEGFP treated cells (bottom). (ANOVA paired tests: *p < 0.05, **p < 0.01)

To figure out the role of CEMIP in OA chondrocyte regulation, we performed Gene Set Enrichment Analysis (GSEA). Both immune/inflammatory systems and the epithelial–mesenchymal transition (EMT) were impaired upon CEMIP depletion (Fig. 3c). Enrichment plots and heatmap of downregulated genes in dedifferentiated chondrocytes treated with shCEMIP #1 and #2 from the EMT dataset are shown on Fig. 4a, left. Decreased expression of mRNA levels of *POSTN* (coding for periostin), *SERPINE1* (coding for PAI-1, a downstream target of TGFβ signaling), *IL32*, *DKK1* and *DKK2* (5 genes implicated in OA pathology)^29–33^ upon both CEMIP deficiency was confirmed on another set of patients by RT-qPCR (Fig. 4a, right). Moreover, at the protein level, the secretion of both periostin and DKK1 was also decreased in CEMIP-depleted chondrocytes compared to shEGFP control cells (Fig. 4b). As CEMIP regulates genes involved in EMT and participated to the expression of two Wnt/β-catenin inhibitors (DKK1 and DKK2), β-catenin expression, also known as an EMT regulator, was assessed. In the RNA-Seq analysis, no variation of *CTNB1* (coding for β-catenin) expression was observed among the different experimental conditions. This was confirmed by RT-qPCR analysis. However, β-catenin protein levels were decreased in CEMIP-depleted cells (Fig. 4c). In parallel, the expression of β-TRCP at mRNA and protein level, a specific E3 ligase of β-catenin, was increased (Fig. 4c). However, there was no modification of Axin-2 expression, another regulator of β-catenin, in CEMIP-depleted cells (data not shown).

### CEMIP as a pro-fibrotic mediator

**Fig. 5:**
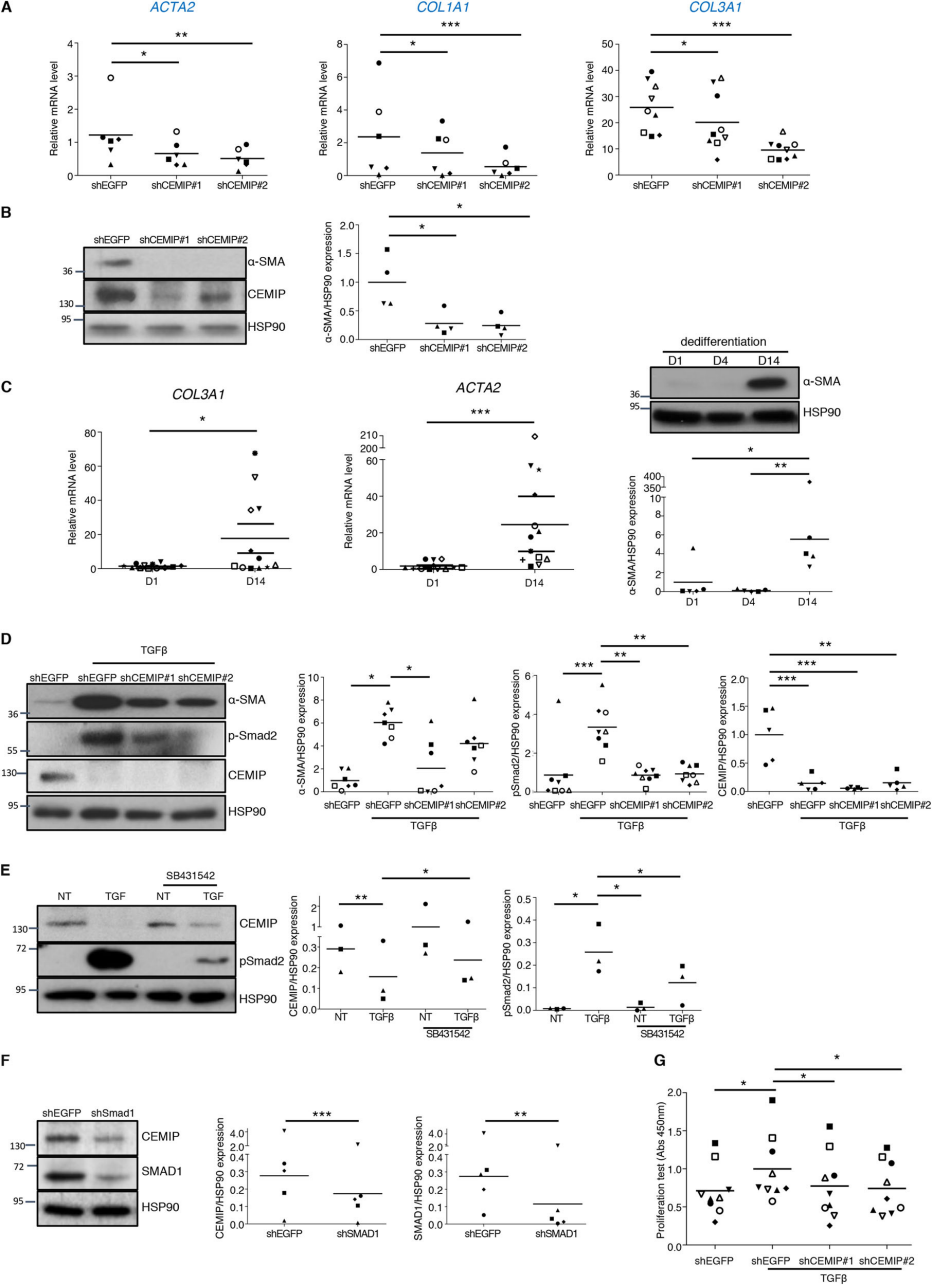
CEMIP induces a fibrosis-like process in human dedifferentiated chondrocytes. **a** RT-qPCR analysis of *ACTA2*, *COL1A1*, and *COL3A1* genes in CEMIP-depleted chondrocytes compared to non-depleted chondrocytes. *ACTA2* (*n* = 6), *COL1A1* (*n* = 6), *COL3A1* (*n* = 9) relative gene expressions were decreased in CEMIP-depleted cells. (ANOVA paired tests: *p < 0.05, **p < 0.01, ***p < 0.001). **b** Representative picture of western blot analysis of α-SMA, CEMIP, and HSP90 upon CEMIP depletion. Analysis was done on several patients (*n* = 4): western blot quantification illustrating the decrease of αSMA/HSP90 expression in CEMIP-depleted cells compared to non-depleted cells. (ANOVA paired tests: *p < 0.05). **c** *COL3A1* (*n* = 12) and *ACTA2* (*n* = 12) mRNA levels were increased at D14 compared to D1 (left). (Error bar: SED, Wilcoxon-paired test: *p < 0.05, ***p < 0.001). Representative picture of western blot analysis of α-SMA and HSP90 at D1, D4, and D14. Analysis was done on several patients (*n* = 5): western blot quantification illustrating the increase of α-SMA/HSP90 expression at D14 compared to D1 and D4 (right). (ANOVA paired tests: *p < 0.05 and **p < 0.01). **d** Representative picture of western blot analysis of α-SMA, p-Smad2, CEMIP, and HSP90 upon CEMIP depletion and TGFβ stimulation. Analysis was done on several patients and western blot quantification illustrating: the increase of αSMA/HSP90 expression upon TGFβ stimulation and its decrease in CEMIP-depleted cells compared to non-depleted cells upon TGFβ stimulation (*n* = 7); the increase of p-Smad2/HSP90 expression upon TGFβ stimulation and its decrease in CEMIP-depleted cells compared to non-depleted cells upon TGFβ stimulation (*n* = 8); the decrease of CEMIP/HSP90 expression upon TGFβ stimulation and in CEMIP-depleted cells compared to non-depleted cells upon TGFβ stimulation (*n* = 5) (right). (ANOVA paired tests: *p < 0.05, **p < 0.01, ***p < 0.001). **e** Representative picture of western blot analysis of CEMIP, p-Smad2, and HSP90 upon SB431542 stimulation. Analysis was done on several patients and western blot quantification illustrating: the decrease of CEMIP/HSP90 expression upon TGFβ stimulation; the increase of CEMIP/HSP90 expression upon SB431542 stimulation in TGFβ treated cells; the increase expression of pSmad2/HSP90 upon TGFβ stimulation; the increase expression of pSmad2/HSP90 upon TGFβ stimulation in SB431542 treated cells; the decrease expression of pSmad2/HSP90 upon SB431542 stimulation in TGFβ treated cells; (*n* = 3) (ANOVA paired tests: *p < 0.05, **p < 0.01). **f** Representative picture of western blot analysis of CEMIP, SMAD1, and HSP90 upon SMAD1 depletion. Analysis was done on several patients and western blot quantification illustrating: the decrease of CEMIP/HSP90 expression in SMAD1 depleted cells and the decrease of SMAD1/HSP90 expression in SMAD1 depleted cells; (*n* = 5) (ANOVA paired tests: **p < 0.01, ***p < 0.001). **g** Graphic representation of BrdU proliferation test. Relative absorbance was increased in cells stimulated with TGFβ compared to non-treated cells and decreased in CEMIP-depleted cells compared to non-depleted cells upon TGFβ stimulation (*n* = 9). (ANOVA paired tests: *p < 0.05)

Pro-fibrotic markers were also detected as down-regulated genes from the EMT dataset of GSEA analysis after CEMIP depletion. The role of CEMIP in fibrosis was therefore studied accordingly. mRNA levels of fibrosis-related *COL1A1*, *COL3A1*, and *ACTA2* (coding for the protein smooth muscle α−2 actin, “α-SMA”) were assessed by RT-qPCR. All levels were decreased in dedifferentiated chondrocytes treated with CEMIP shRNAs compared to control EGFP shRNA (Fig. 5a). Moreover, α-SMA expression was also decreased at the protein level in CEMIP-depleted cells (Fig. 5b). Recently, we highlighted that *COL1A1* mRNA level was increased in dedifferentiated chondrocytes compared to freshly isolated chondrocytes^17^ (see also supplementary data 1C). Here, *COL3A1* and *ACTA2* mRNA as well as its protein α-SMA also increased along chondrocytes dedifferentiation (Fig. 5c). As TGFβ signaling is a well-known inducer of cell proliferation and articular fibrosis^34^, this pathway was investigated in the regulation of CEMIP. Upon TGFβ stimulation, CEMIP expression was decreased, α-SMA and p-Smad2 were increased (Fig. 5d), and pSmad1/5 was not modified (Supplementary data 3). Moreover, CEMIP deficiency interfered with the induction of α-SMA expression as well as with Smad2 activation upon stimulation with TGFβ but did not modified pSmad1/5 expression. Inhibition of p-Smad2/3 (Alk5/PAI-1) pathway of the TGFβ signaling by using the specific inhibitor SB431542 increased CEMIP expression and as expected, inactivated Smad2 in TGFβ-stimulated chondrocytes (Fig. 5e). Moreover, inhibition of pSmad1/5 (Alk1) pathway of the TGFβ signaling using shRNA directed against *SMAD1* led to a decrease of CEMIP expression (Fig. 5f). Finally, the induction of dedifferentiated chondrocytes proliferation by TGFβ was severely impaired upon CEMIP deficiency (Fig. 5g). Of note, we did not detect any effect on dedifferentiated chondrocytes viability after CEMIP depletion (data not shown).

All these data unravel an important role for CEMIP in chondrocytes proliferation and as a pro-fibrotic mediator.

### In situ expression of fibrosis markers

**Fig. 6:**
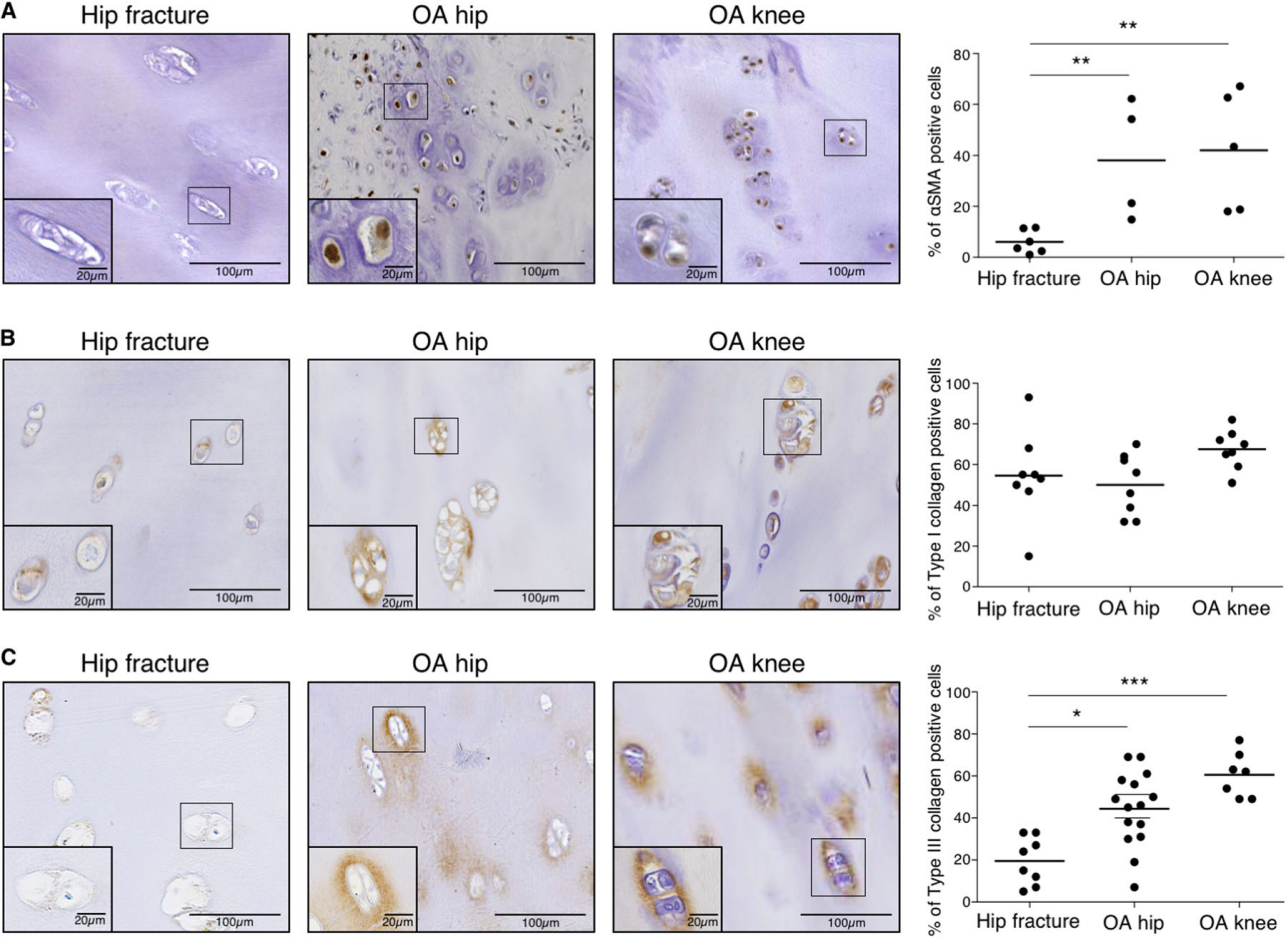
αSMA and type III collagen (but not type I) expression are increased in human OA cartilage. **a** Representative picture of IHC analysis of cartilage section from human hip fracture, OA hip and OA knee stained with anti-αSMA antibody. Analysis was done on several patients: the percentage of αSMA positive cells was significantly increased in OA hip (*n* = 4) and OA knee (*n* = 5) compared to hip fracture (*n* = 6). (ANOVA tests: **p < 0.01). **b** Representative picture of IHC analysis of cartilage section from human hip fracture, OA hip and OA knee stained with anti-type I collagen antibody. Analysis was done on several patients: the percentage of type I collagen positive cells was not significantly different in OA hip (*n* = 8) and OA knee (*n* = 8) compared to hip fracture (*n* = 8). (ANOVA tests: not significant for both comparisons). **c** Representative picture of IHC analysis of cartilage section from human hip fracture, OA hip and OA knee stained with anti-type III collagen antibody. Analysis was done on several patients: the percentage of type III collagen positive cells was significantly increased in OA hip (*n* = 15) and OA knee (*n* = 7) compared to hip fracture (*n* = 8). (Error bar: SED, ANOVA tests: *p < 0.05, ***p < 0.001)

To see whether fibrosis could be observed in situ, α-SMA and type I and III collagen expressions in human cartilage were investigated by IHC. IHC quantification demonstrated that α-SMA and type III (but not type I) collagen expressions were significantly increased in OA human hip and knee compared to healthy human hip cartilage (Fig. 6). Representative images of IHC negative controls are shown in Supplementary data 4.

### In situ characterization of CEMIP expressing chondrocytes

**Fig. 7:**
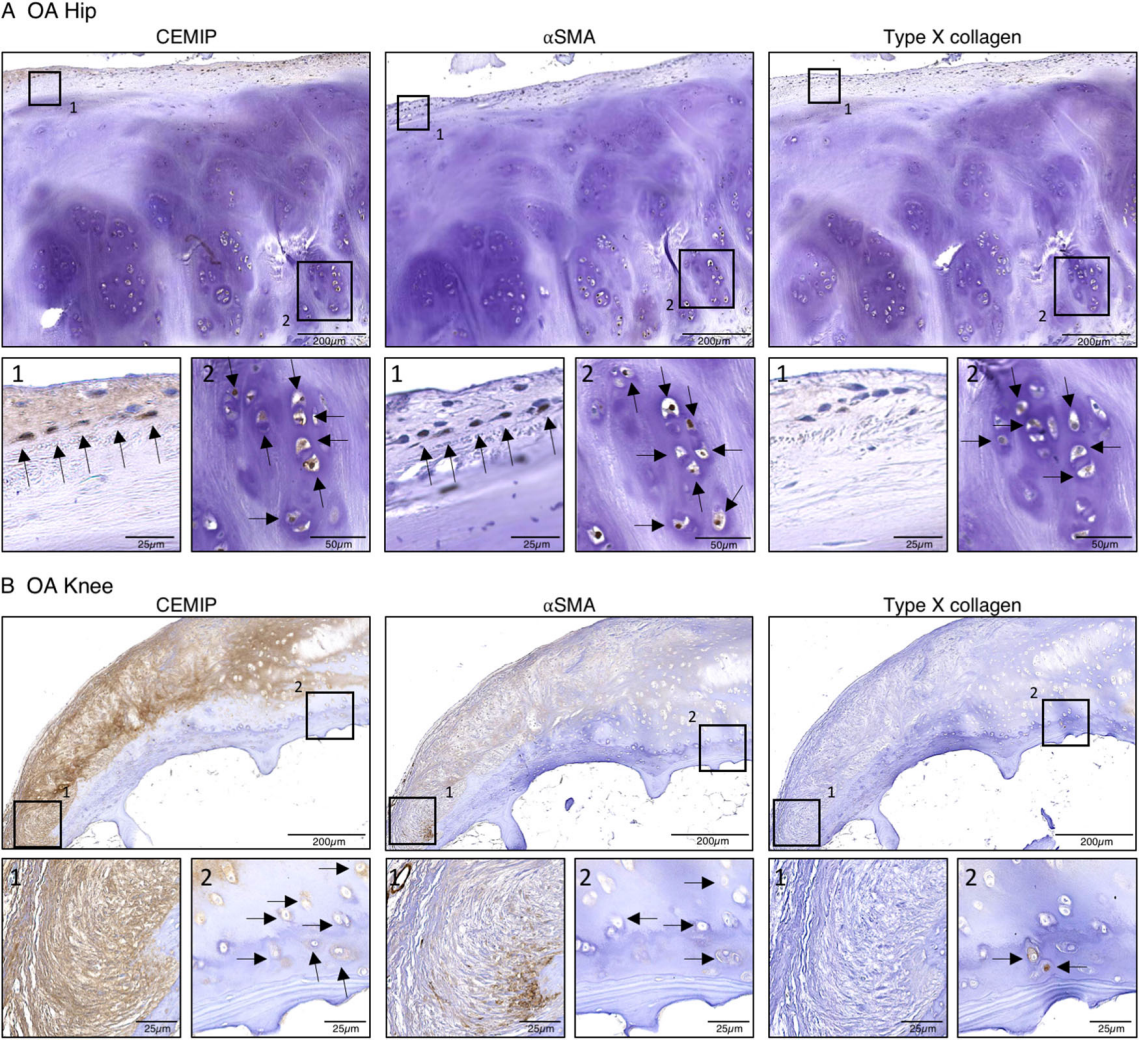
CEMIP is co-expressed with αSMA in the upper layer and with αSMA and type X collagen the deep zone of human OA cartilage. **a** Representative picture of IHC analysis (*n* = 2) of cartilage section from OA hip stained with anti-CEMIP (left), anti-αSMA (middle), and anti-Type X collagen (right) antibodies. **b** Representative picture of IHC analysis (*n* = 4) of cartilage section from OA knee stained with anti-CEMIP (left), anti-αSMA (middle), and anti-Type X collagen (right) antibodies

To better characterize chondrocytes expressing CEMIP in OA cartilage, immunodetection of CEMIP, αSMA (a marker of fibrosis), and type X collagen (an hypertrophic chondrocyte marker) were performed on serial tissue sections provided from the same OA hip or knee sample. CEMIP and αSMA were both expressed in the upper and the deep zone of OA cartilage section. By contrast, type X collagen was rather expressed in the deep and the calcified zone (Fig. 7). OA chondrocytes expressing CEMIP and αSMA in the upper zone appeared elongated while OA chondrocytes expressing CEMIP, αSMA, and type X collagen in the deep zone appeared round and in clusters.

## Discussion

CEMIP is a protein of emerging interest. Many studies have shown that it plays a critical role in cancer cell survival, proliferation and migration, three critical processes involved in cancer development and dissemination^5,6^. Interestingly, Yoshida and co-workers have highlighted that CEMIP was implicated in hyaluronic acid turnover and was increased in synovial fibroblasts from patients with OA and RA. Using KO mice, Shimoda et al.^10^ have shown that CEMIP controls the endochondral ossification.

In this work, we pointed out that CEMIP was overexpressed in human and mouse OA cartilage compared to healthy cartilage. Interestingly, in mouse OA cartilage, we observed that CEMIP overexpression decreased in the late stages of the disease. These data suggest that CEMIP could have a significant role in the molecular disturbance encountered at an early stage of the disease. In accordance, very recently Shimizu et al.^35^, showed that CEMIP expression correlated with Mankin score of OA patients.

Molecular mechanisms that lead to OA are still largely unknown. However, it is likely that aberrant activity of OA chondrocytes leads to cartilage ECM remodeling^36^. Our results indicate that CEMIP induced a variation in the expression panel of collagen types. Indeed, human chondrocytes expressing CEMIP produced more type I, III, IV and V collagen mRNAs and less type II, VI, IX, and XI than chondrocytes where CEMIP expression is abrogated. Modulation of collagen expression pattern by chondrocytes occurred in line with a transition to a fibroblastic cell shape called “dedifferentiation”^11^. Recently, we highlighted that dedifferentiated chondrocytes were able to express type I collagen, and inversely that type II and X collagen were expressed by freshly isolated chondrocytes but nearly not by dedifferentiated chondrocytes^17^. Here, we show that CEMIP is lightly expressed in freshly isolated OA chondrocytes expressing type X collagen, but is increased at an early stage of chondrocyte dedifferentiation (day 4) and before any other dedifferentiated chondrocytes markers (type I and III collagen, β-Catenin, and αSMA). All these data suggest that CEMIP could be an inducer of human chondrocyte dedifferentiation process leading to a fibroblastic-like phenotype.

This finding is supported by the role of CEMIP in the regulation of genes involved in mesenchymal transition process. Indeed, we showed that CEMIP depletion induced a modulation of genes belonging to the Hallmark_Epithelial_Mesenchymal_Transition pathway (GSEA analysis) with, among others, a decreased periostin secretion (and POSTN mRNA). As a ligand of α_V_β_3_ and α_V_β_5_ integrins, periostin fostered adhesion and migration of epithelial cells^37^. Moreover, periostin is upregulated in OA cartilage and intensifies inflammation and metalloproteinases production^38,39^. We also showed that CEMIP regulated *SERPINE1* gene coding for PAI-1. PAI-1 is a downstream target of the pro-fibrotic side of the TGFβ signaling (p-Smad2/3 (Alk5-PAI-1))^30^. It also has an important role in fibrinolysis and thrombosis^40^. Our results are in accordance with the literature in which the role of CEMIP in the EMT process is observed in different cell types^6,41,42^. We also found that CEMIP modulated the Wnt/β-catenin signaling, a well-known regulator of EMT, in human chondrocytes. The Wnt/β-catenin pathway contributes to OA pathogenesis^43,44^. We previously observed that β-catenin accumulated along chondrocytes dedifferentiation leading to leptin production^17^. Here, we detected that CEMIP expression also increased with chondrocytes dedifferentiation. Besides, we observed a decrease of β-catenin protein level (but not mRNA) in CEMIP-depleted chondrocytes. This could indicate that CEMIP prevented β-catenin degradation by reducing β-TRCP expression (a β-catenin-specific E3 ligase). Our results are in line with findings on gastric and colon cancer cells in which CEMIP activate the Wnt/β-catenin pathway^3,41^. Inversely, β-catenin silencing induces a decrease of CEMIP expression leading to a positive feedback loop in the regulation of β-catenin expression^7^.

β-catenin pathway also appears to be implicated in fibrosis^45,46^. Growing evidences suggest a cross-talk between Wnt/β-catenin and TGFβ pathway inducing a pro-fibrotic process^45,47^. In this work, we observed that CEMIP induced a fibrosis-like process in OA chondrocyte. Indeed, additionally to its role in the Wnt/β-catenin pathway activation and in the induction of types I and III collagen expression, we showed that αSMA expression was sharply diminished with the decrease of CEMIP level even upon TGFβ induction. Furthermore, the abrogation of CEMIP expression led to the decrease of p-Smad2 expression induced by TGFβ and the decrease of *SERPINE1* gene (coding for PAI-1) expression. This indicated that CEMIP promoted the TGFβ signaling towards its pro-fibrotic side (p-Smad2/3 (Alk5/PAI-1)). Moreover, TGFβ stimulation led to the activation of p-Smad2/3 (Alk5/PAI-1) pathway but not of pSmad1/5 (Alk1) pathway, and to the inhibition of CEMIP expression. Inactivation of p-Smad2/3 (Alk5/PAI-1) pathway by SB431542 revealed an increase of CEMIP level even after TGFβ stimulation. Additionally, the inhibition of SMAD1 leading to the inactivation of pSmad1/5 (Alk1) pathway induced a decrease of CEMIP expression. These data revealed that CEMIP is inhibited by the activation of p-Smad2/3 (Alk5/PAI-1) triggered by TGFβ whereas activated by the pSmad1/5 (Alk1) pathway. Interestingly, we previously demonstrated that pSmad1/5 (Alk1) pathway is activated in dedifferentiated chondrocytes, conversely to p-Smad2/3 (Alk5/PAI-1) which is activated in freshly isolated chondrocytes^17^. This suggests that the increase expression of CEMIP along chondrocytes dedifferentiation could be induced by pSmad1/5 (Alk1) pathway activation. Of note, the activation of fibrosis pathway, p-Smad2/3(Alk5/PAI-1), by CEMIP seemed to be independent of TGFβ. Indeed, CEMIP deficiency allowed the increase expression of *TGFB1* and *2* mRNA levels (RNA-Seq analysis) suggesting that CEMIP is a downregulator of TGFβ. Finally, we demonstrated that CEMIP is essential for chondrocytes proliferation (but not viability) triggered by TGFβ. This sustains the hypothesis that CEMIP is a cell proliferation inducer^5,6^.

In the same way as CEMIP, we observed that *COL3A1* and *ACTA2* (as well as αSMA protein) were not expressed in freshly isolated chondrocytes but largely expressed in dedifferentiated chondrocytes after 14 days in culture. This suggests that dedifferentiated chondrocytes shared expression pattern with activated myofibroblasts (namely “chondro-myo-fibroblasts”). Kinner et al.^48^ have also observed the lack of αSMA expression in freshly isolated chondrocytes and have observed an increase of expression with cell passage number. Moreover, like CEMIP, αSMA, and type III collagen were weakly expressed in healthy tissue and largely expressed in OA cartilage. Recently, Hosseininia and co-worker have underscored the increase expression of type III collagen in OA hip cartilage compared to healthy hip cartilage^49^. Here, we validated that type III collagen was significantly increased in OA hip cartilage but also in OA knee cartilage compared to non-OA cartilage. Moreover, we showed that type I collagen is expressed in healthy cartilage and that its expression remained constant in OA hip and knee. This differs from the results of Aigner and colleagues who did not detect mRNA and a very low level of protein of type I collagen in the upper layer of cartilage^50^.

Both in vitro and in situ, CEMIP expression seems to be concomitantly expressed with the fibrosis marker αSMA. Indeed, in the same way than αSMA, CEMIP expression increased along chondrocyte dedifferentiation and in OA cartilage compared to healthy cartilage. However, in OA articular cartilage, chondrocytes are reported to express type X collagen, a marker of hypertrophic chondrocytes^51^. In situ characterization of CEMIP expressing chondrocytes showed that CEMIP was co-expressed with αSMA in all layers of OA cartilage and co-expressed with type X collagen only in the deep zone. This means that different chondrocyte phenotypes coexist in situ in osteoarthritic cartilage: on one hand, elongated chondrocytes expressing CEMIP and αSMA are in the upper part of OA cartilage, and on the other hand clusters of chondrocytes expressing CEMIP, αSMA, and type X collagen are in the lower part of OA cartilage. These results strengthen our postulate that CEMIP regulated chondro-myo-fibroblasts expressing αSMA. The role of CEMIP in hypertrophic chondrocytes is suggested by its co-expression with type X collagen. Nevertheless these hypertrophic chondrocytes expressed concomitantly αSMA. In addition, type X collagen was present in freshly isolated chondrocyte but not in dedifferentiated chondrocytes, conversely to CEMIP^17^.

Fibrosis is an unresolved wound-healing process characterized by excessive deposition of extracellular matrix components and cell proliferation leading to disrupted tissue function^52^. The unresolved wound healing is ensured by myofibroblasts expressing αSMA, which gives them contractility properties. In OA, it is well known that fibrosis occurs in synovium^53^. Moreover, macroscopic features of fibrosis have been identified in the cartilage of DMM mice^54^. We showed that αSMA, type III collagen (two fibrosis markers) and CEMIP (that we unraveled as a fibrosis regulator) were weakly expressed in healthy tissue and largely expressed in OA cartilage from hip and knee, which suggests that unresolved wound healing is occurring in OA cartilage and that chondrocytes have acquired new functions shared by activated myofibroblasts (“chondro-myo-fibroblasts”). It is likely that cartilage fibrosis occur in parallel with chondrocytes proliferation encountered in early stages of the pathology.

**Fig. 8:**
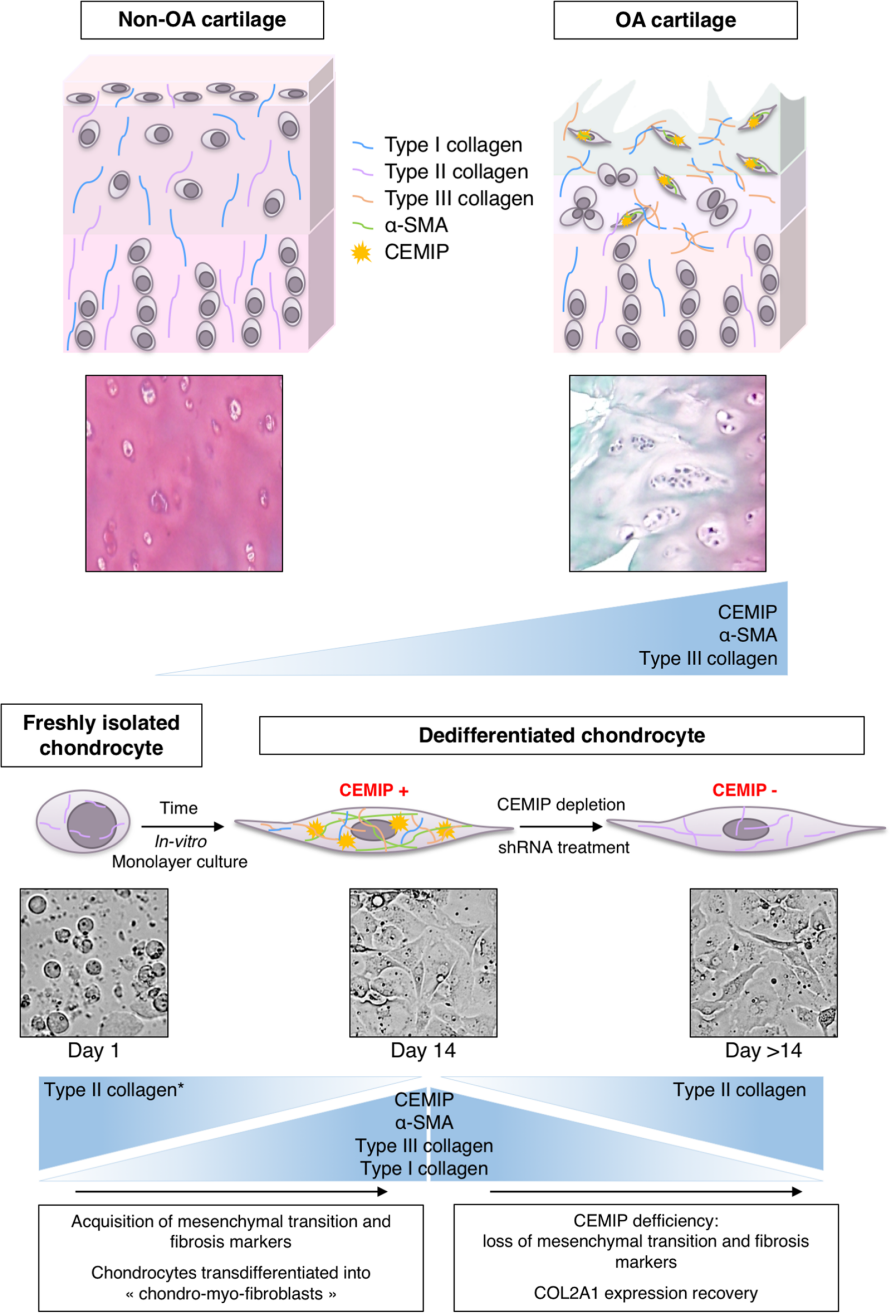
Schematic representation of CEMIP-, αSMA-, and collagen-type expression. CEMIP, αSMA, and type III collagen are overexpressed in OA cartilage compared to non-OA cartilage (upper panel). CEMIP, αSMA, and types I and III collagen are overexpressed in dedifferentiated chondrocytes compared to freshly isolated chondrocytes. All these decreased when CEMIP expression is abolished. Type II collagen expression decreased along chondrocytes dedifferentiation^17^ and increased while CEMIP expression is abolished (lower panel)

Our findings suggest that CEMIP is sharply increased in OA cartilage and along chondrocytes dedifferentiation. In vitro, CEMIP regulated proliferation and transdifferentiation of chondrocytes into “chondro-myo-fibroblasts” expressing α-SMA and COL3A1, two fibrosis markers. Moreover, these “chondro-myo-fibroblasts” (expressing CEMIP, α-SMA and type III collagen) are found in situ in OA cartilage but not in healthy cartilage revealing a fibrosis process during OA (Fig. 8). As CEMIP seems to be an early actor in the deregulation leading to OA, deciphering its precise role in cartilage remodeling using a KO mice model should be considered. Moreover, it could be interesting to investigate the role of CEMIP in other arthrofibrosis conditions known and other pathologies for which, in the long term, fibrosis leads to the impairing of proper organ function.

## Supplementary information

Supplementary Data 1

Supplementary Data 2

Supplementary Data 3

Supplementary Data 4
