# Supplementary figures and images for: CEMIP (KIAA1199) induces a fibrosis-like process in osteoarthritic chondrocytes
Source: Cell Death Dis. 2019 Feb 4;10(2):103. doi: 10.1038/s41419-019-1377-8 (PMC6362103; doi:10.1038/s41419-019-1377-8)

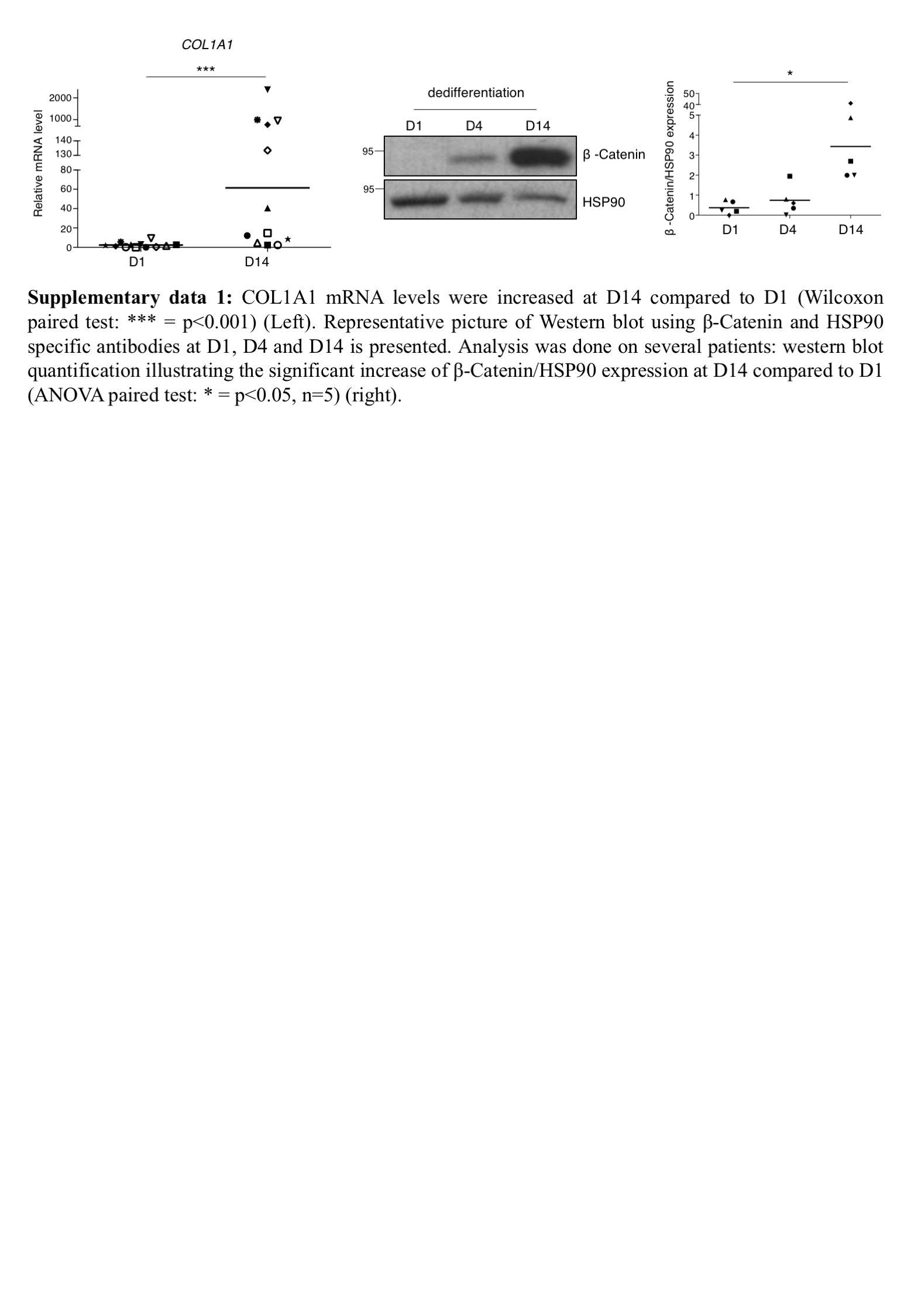

Supplement: Supplementary file 1 — Supplementary Data 1 [file 41419_2019_1377_MOESM1_ESM.tif]

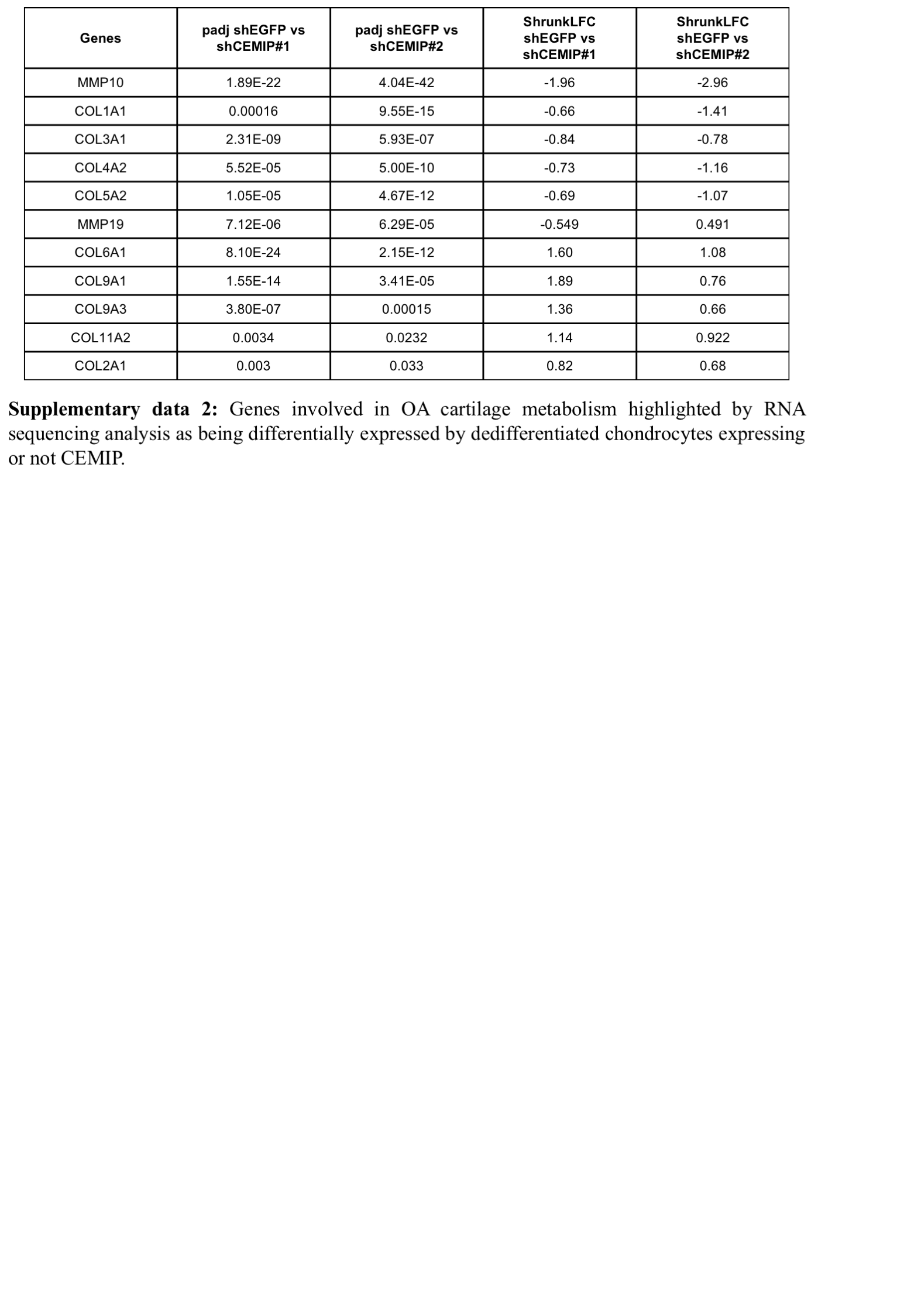

Supplement: Supplementary file 2 — Supplementary Data 2 [file 41419_2019_1377_MOESM2_ESM.tif]

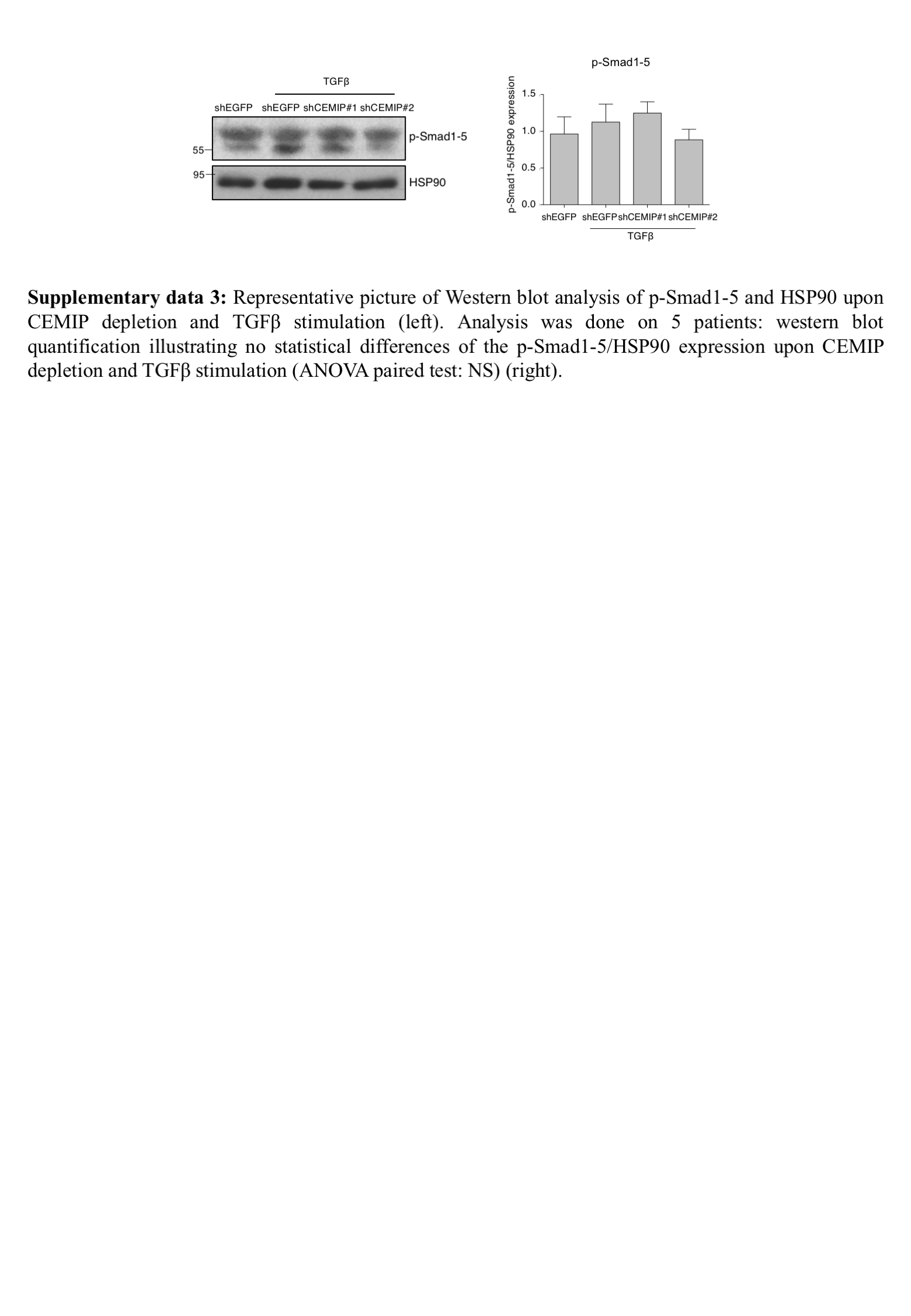

Supplement: Supplementary file 3 — Supplementary Data 3 [file 41419_2019_1377_MOESM3_ESM.tif]

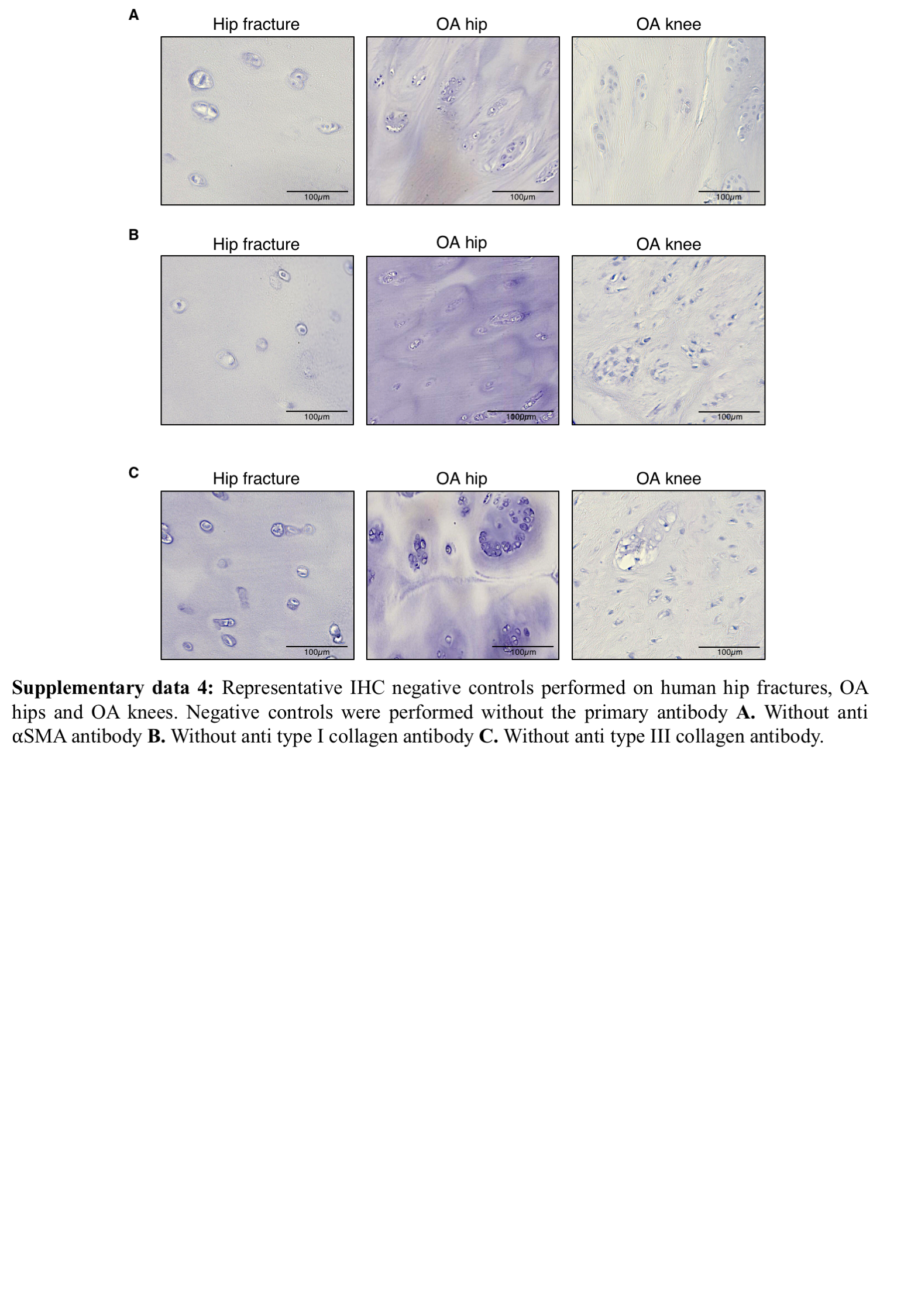

Supplement: Supplementary file 4 — Supplementary Data 4 [file 41419_2019_1377_MOESM4_ESM.tif]
